# Stabilization of H_3_^+^ in the high pressure crystalline structure of H_*n*_Cl (*n* = 2–7)

**DOI:** 10.1039/c4sc02802c

**Published:** 2014-10-21

**Authors:** Ziwei Wang, Hui Wang, John S. Tse, Toshiaki Iitaka, Yanming Ma

**Affiliations:** a State Key Laboratory of Superhard Materials , Jilin University , Changchun 130012 , China . Email: huiwang@jlu.edu.cn; b Department of Physics and Engineering Physics , University of Saskatchewan , Saskatoon , S7N 5B2 , Canada . Email: john.tse@usask.ca; c Beijing Computational Science Research Center , Beijing 10084 , China; d Computational Astrophysics Laboratory , RIKEN , 2-1 Hirosawa , Wako , Saitama 351-0198 , Japan

## Abstract

The particle-swarm optimization method has been used to predict the stable high pressure structures up to 300 GPa of hydrogen-rich group 17 chlorine (H_*n*_Cl, *n* = 2–7) compounds. In comparison to the group 1 and 2 hydrides, the structural modification associated with increasing pressure and hydrogen concentration is much less dramatic. The polymeric HCl chains already present in the low temperature phase under ambient pressure persist in all the high pressure structures. No transfer of electrons from the chlorine atoms into the interstitial sites is found. This indicates the chemical bonding at high pressure in group 17 elements is fundamentally different from the alkali and alkaline elements. It is found that almost perfectly triangular H_3_^+^ ions can be stabilized in the crystalline structure of H_5_Cl.

## Abstract 2

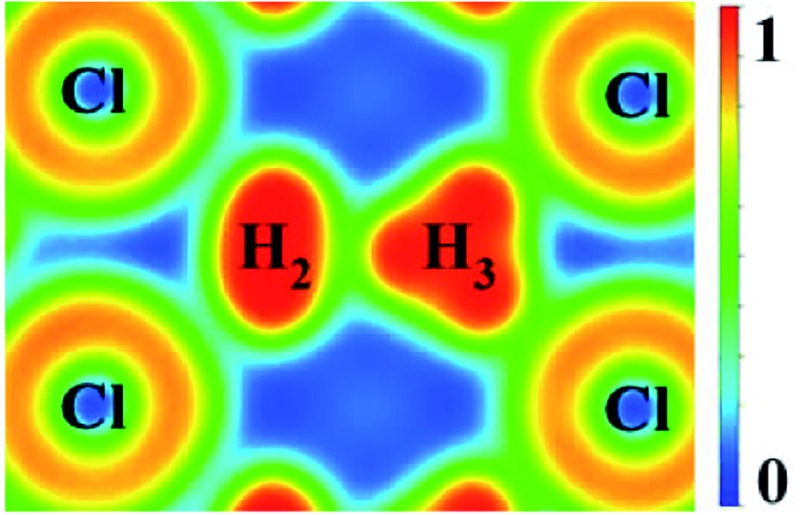
Using the CALYPSO structure searching method, multicenter bonding H_3_^+^ ions were stabilized under high pressure in a hydrogen rich H–Cl system.

## Introduction

The suggestion that the hydrides of main group elements at high pressure may be superconductors with high critical temperature has stimulated recent interest in the search for the possible existence of hydrogen-rich alloys by theoretical and experimental means.^1^ The former has been greatly facilitated by the recent developments in practical strategies for the prediction of crystal structures. Although, the predicted hydrides have yet to be confirmed by experiment, the theoretical studies have revealed a myriad of novel structures for the high pressure hydrides and enriched the understanding of the nature of chemical bonding in the rarely explored high pressure regime.

The hydrides of group 1 and 2 elements formed from the reaction of the metal and hydrogen molecules have been the most studied.^2–6^ Most of the structures and structural trends can be explained from the simple concept of electron transfer from the metal to the hydrogen due to the large electronegativity differences between the alkali and alkaline elements and hydrogen molecules. The predicted compounds display rich H species distinguished from the well-known H-ion in hydrides at traditional stoichiometric ratios. Perhaps one of the most exciting predictions is the emergence of symmetric and linear H_3_^–^ at high pressures as observed in dense CsH_3_ and BaH_6_.^7,8^ Moreover, the formation pressures of these compounds of just a few tens of GPa are accessible by experiments. In comparison, the bonding pattern is quite different for group 14 and transition elements. For example, a Van der Waals solid with such molecular H_2_ units was found experimentally in SiH_4_ at low pressure.^9^ At higher pressure, the atoms of group 14 elements tend to aggregate to form a 2D layered structure decorated with molecular like H_2_ species as predicted for SiH_4_ and SnH_4_.^10,11^ In comparison, the high pressure chemistry of hydrogen with electron-rich group 17 halogens has not been investigated. In this paper, we present results on a study of the crystal structures and phase stabilities of hydrogen-rich HCl–H_2_ system. A major finding is the stabilization of cationic (H_3_)^+^ (H_2_) species in H_5_Cl. The geometry of H_3_^+^ becomes almost an equilateral triangle under very high pressure.

The observation of a triatomic hydrogen cation H_3_^+^ in the solid state is new and significant. The isolated molecule is important in various branches of science, such as physics, chemistry and astronomy. For example, it is known that the H_3_^+^ ion with a triangular configuration is stable in the interstellar medium thanks to the low temperature and low density of the interstellar space and the H_3_ molecule is commonly formed from the neutralization reaction of H_3_^+^ and an electron, and rather evanescent as a result of the repulsive nature of its ground state.^12^ Furthermore, the multicenter (H_3_)^+^ (H_2_) bond in group 17 Cl compounds signifies a deviation in the nature of chemical bonding from the charge transfer interactions in group 1 and 2 hydrides and covalent bonding in group 14 hydrides.

## Computational details

The search for stable high pressure structures of the H_*n*_Cl system was based on global minimization of free energy surfaces using *ab initio* total energy calculations and the particle–swarm-optimization scheme as implemented in the CALYPSO (Crystal structure AnaLYsis by Particle Swarm Optimization) code.^13,14^ The performance and reliability of this method has been demonstrated on many known systems. An example is the success on the prediction^15–17^ of an insulating orthorhombic (*Aba*2, Pearson symbol o*C*40) structure of Li and, more recently, two low-pressure monoclinic structures of Bi_2_ Te_3_. In both cases, the predicted structures were later confirmed by experiments.^18,19^ Structural searching was performed at 100, 200, and 300 GPa with a simulation cell consisting of 1–4 formula units. *Ab initio* electronic structure calculations and structural relaxations were carried out using density functional theory with the Perdew–Burke–Ernzerhof (PBE) exchange-correlation^20^ implemented in the Vienna *ab initio* Simulation Package (VASP) code.^21^ The predicted stable structures were carefully optimized at a high level of accuracy. A plane wave energy cutoff of 1000 eV was employed. Large Monkhorst-Pack *k* point sampling grids^22^ were used to ensure that all the enthalpy calculations were well converged to an accuracy of 1 meV per atom. The atomic charges were obtained from Bader topological analysis^23–25^ with very large grids to ensure sufficient accuracy. We also performed additional calculations employing the DF-2 van der Waals (vdW) functional^26^ to validate the results, in particular for the low pressure structures. Phonons were calculated with the supercell method^27^ implemented in the PHONOPY program.^28^ In essence, from finite displacements, the Hellmann–Feynman atomic forces computed at the optimized supercell by the VASP code were input to the PHONOPY code to construct the dynamical matrix. Diagonalization of the dynamical matrix gives the normal modes and their frequencies. Converged results were obtained with the use of a 2 × 2 × 1 supercell and 4 × 4 × 6 *k*-meshes for the *Cc* structure, and a 2 × 2 × 1 supercell and 4 × 4 × 6 *k*-meshes for the *C*2/*c* structure.

## Results and discussion

**Fig. 1:**
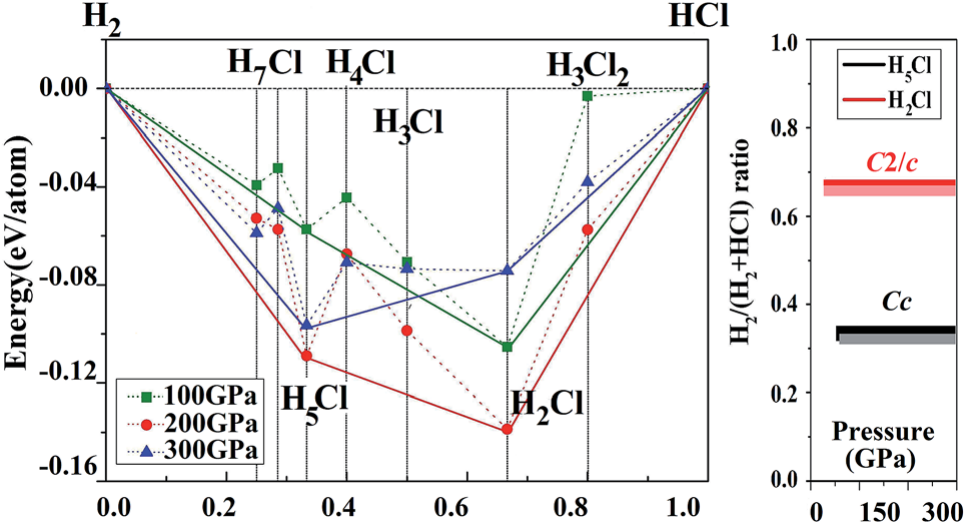
Stability of new hydrogen chlorides. (A) Enthalpies of formation (ΔH, with respect to HCl and H_2_ of their most stable phases at selected pressures) of H_*n*_Cl (*n* = 1–7). The abscissa *x* is the fraction of H_2_ in the structures. Circles on the solid lines represent stable ground-state compounds under the corresponding pressure. (B) Pressure-composition phase diagram of the H–Cl system. The lighter colored lines represent the vdW corrections to corresponding structures.

Before embarking on a detailed discussion of the structures of the predicted high pressure H_*n*_Cl polymers, the relative energetics of the H–Cl system with H-rich stoichiometry from 100 to 300 GPa are summarized in the convex hull plot shown in Fig. 1. The enthalpies of formation were evaluated as the difference in the enthalpy of the predicted H_*n*_Cl structure with solid HCl and H_2_ at the selected pressures. Since hydrogen has a small atomic mass, the zero point energy (ZPE) may be important. To investigate the vibrational effects on the phase stability, ZPEs for H_2_Cl and H_5_Cl were estimated at 100–300 GPa from the corresponding phonon spectra using the quasi-harmonic approximation.^29^ It is found that the ZPEs are quite small and the inclusion of ZPEs in the phase diagram only resulted in a slight shift in the formation pressures but the stability of both phases remains unaltered. Structures lying on the convex hull are thermodynamically stable or metastable and, in principle, can be synthesized. Fig. 1 reveals that only two HCl–H_2_ complexes, H_2_Cl and H_5_Cl are stable at 100 GPa. At this pressure H_2_Cl has the most negative enthalpy of formation. With increasing pressure, the stability of H_5_Cl relative to H_2_Cl increases and becomes the most stable phase at 300 GPa.

vdW effects may play an important role in the stabilization of a molecular solid. We have thus performed additional calculations on the H–Cl system with the vdW-DF2 method.^26^ The results show that the differences between calculations with and without vdW corrections on the formation enthalpies of the structures considered in Fig. 1 are small. The formation pressures were found to change slightly. For example, the stabilized pressure of H_2_Cl increased from 21.2 to 21.3 GPa, while for H_5_Cl it increased from 50 to 60 GPa. Otherwise, the energetic order remains the same.

**Fig. 2:**
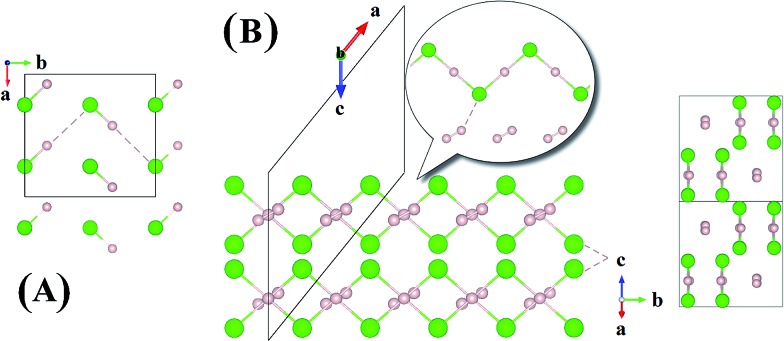
Crystal structures of hydrogen chlorides. (A) Experimental structure of HCl at ambient pressure and low temperature, (B) *C*2/*c*-H_2_Cl recovered at 100 GPa along a different angle.

Now we examine the development of the high pressure crystal structures in H_*n*_Cl (*n* = 2–7). The starting point is the crystal structure of HCl under ambient pressure. At low temperature, X-ray and neutron diffraction show HCl crystallized in an orthorhombic structure (*Bb*2_1_ *m*).^30^ In the crystal, HCl molecules are linked *via* the H atoms forming zigzag chains running parallel to the crystallographic *b* axis. The nearest neighbour Cl–H and second nearest neighbour Cl···H distances are 1.25 Å and 2.44 Å, respectively. The Cl···Cl separation is 3.88 Å and the H–Cl···H valence angle is 93.6°. The predicted crystalline phase of H_2_Cl at 100 GPa has a *C*2/*c* space group and the structure is shown in Fig. 2. The crystal is formed from HCl chains interposed with H_2_ molecules. In this case, the H in the HCl chain is midway between the two Cl atoms with an H–Cl distance of 1.45 Å. The H–Cl–H angle has opened to 97.9° and the Cl···Cl separation is shortened to 2.90 Å. The H_2_ units in the structure all have a H–H distance of 0.74 Å, which is almost identical to that of the isolated molecule. The Bader charges for the H in the chain and Cl atoms are +0.44 and –0.35, respectively and 0.0 for the H atoms in the H_2_ units. The closest contact between a Cl atom and the H_2_ molecule is 1.98 Å. The crystal structure of H_2_Cl at 300 GPa differs little from that at 100 GPa. The H atoms in the H–Cl chains are still situated at the middle of the two neighbouring Cl atoms with a H–Cl distance of 1.35 Å. The H–Cl–H angle is 95.5° and the shortest separation between two Cl atoms has reduced further to 2.69 Å. The H–H bond length in the H_2_ unit is 0.73 Å. The closest H_2_···Cl distance is. 1.70 Å. Compression has a significant effect on the interatomic distances of the H–Cl chains but does not alter fundamentally the underlying bonding pattern. A longer H–Cl distance in the chain suggests increased ionicity of the Cl–H bonds.

**Fig. 3:**
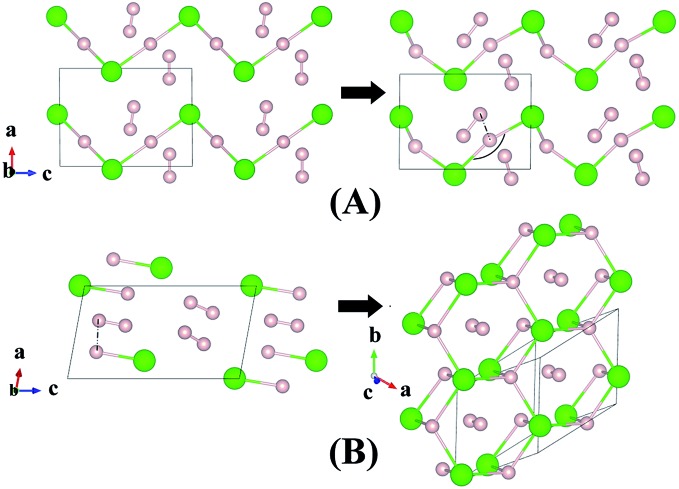
Crystal structures of hydrogen chlorides. Predicted metastable phase H_3_Cl (A) and H_4_Cl (B) at pressures of 100 to 300 GPa.

Although H_3_Cl and H_4_Cl are only metastable, it is instructive to examine the evolution of the crystal structure with increasing H_2_ concentration. The structures of H_3_Cl at 100 and 300 GPa are shown in Fig. 3. Both are composed of zigzag H–Cl chains. Like H_2_Cl, the H atom is equidistant from the two nearest Cl atom with H–Cl bond distances of 1.44 Å at 100 GPa and 1.43 Å at 300 GPa. The most significant difference between the low and high pressure structures is that the Cl–H–Cl angle is almost linear at 100 GPa but bends to 135° at 300 GPa. At 300 GPa, the closest contact between the H_2_ and the H in the chain is 1.27 Å. However, in both cases, the H–H distance of the H_2_ molecule remains 0.73 Å. The structure of H_4_Cl at 100 GPa differs dramatically from all the structures within this series of compounds. Instead of H–Cl chains, the structure is composed of isolated HCl and H_2_ molecules. The H–Cl distance is 1.38 Å and the H–H bond length is 0.74 Å. For comparison, the H–Cl bond of a free molecule is 1.276 Å. Therefore, the distance in the solid state at 100 GPa is slightly longer. The structure of H_4_Cl at 300 GPa again is different from that at 100 GPa. The basic building units are isolated Cl atoms, H_2_ molecules with H–H distance of 0.74 Å and a novel 2-D layer of slightly puckered fused hexagonal rings formed from 3 HCl units with additional H atoms attached to the Cl atoms. Each H atom in the ring is bonded to three Cl atoms. In addition, each Cl is bonded to an extra H atom which is not coordinated to other species in the crystal. The H–Cl distances in the fused ring are 1.59 Å and the terminal H–Cl is substantially shorter at 1.49 Å. Interestingly, the terminal H–Cl–H (ring) angles are 77° and the in-plane H–Cl–H and Cl–H–Cl angles are between 114–115°.

An interesting structure was observed in H_5_Cl at 100 GPa. Although chains formed from Cl and H atoms are still clearly visible, the detailed construction of the chain is very different. In H_5_Cl, instead of placing one H atom midway between the two nearest neighbour Cl atoms, it is replaced by an H_3_ unit. The H_3_ is a distorted isosceles triangle and can be described as a loosely bound unit of an H atom and an elongated H_2_ with an apical angle of 63.8°. The apical H atom is linked to the two nearest Cl atoms in the chain with H–Cl distances of 1.47 Å. The distances from the apical H atom to the two H forming the H_2_ are 1.01 Å and 0.97 Å, respectively and the intermolecular H–H distance is 0.81 Å. The remaining H_2_ units in the structure have H–H distances of 0.74 Å. Moreover, the shortest distance from these H_2_ to the H_3_ is 1.36 Å and, therefore, may be considered as non-interacting molecules.

**Fig. 4:**
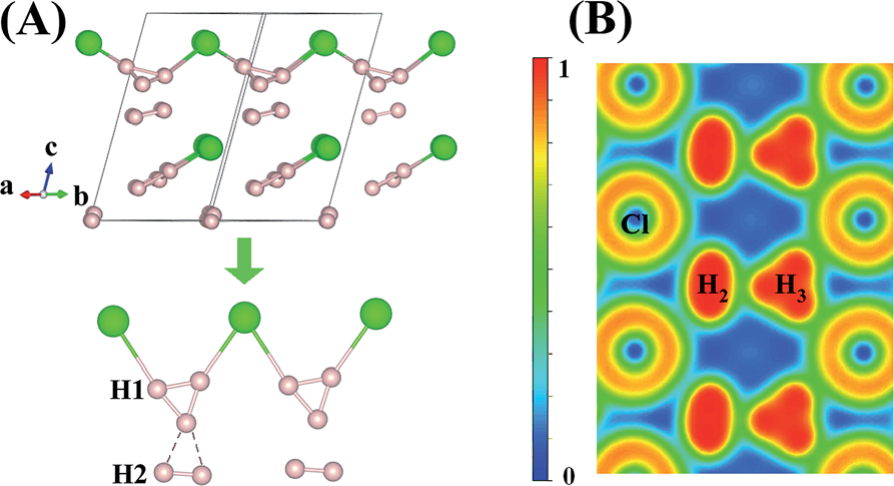
(A) crystal structure of *Cc*-H_5_Cl recovered at 100 GPa and its chain style when compressed to 300 GPa. (B) Electron localization function (ELF) maps of the planes where the hydrogen and chloride atoms lie for the *Cc* structure at 300 GPa.

Compression of H_5_Cl to 300 GPa does not change the space group symmetry. The chain pattern with interpose H_3_ units is still maintained, but the local H···H interactions have changed dramatically. The H_3_ unit now approaches an equilateral triangle. The H–H lengths are 0.87, 0.87 and 0.88 Å with bond angles 59.7, 59.7 and 60.5°. The Cl–H distance has elongated from 1.47 Å at 100 GPa to 1.60 Å! The large lengthening of the Cl–H clearly suggests a substantial change in the Cl–H bonds. More significantly, the isolated H_2_ molecules are now pushed towards the H_3_ units and interact with one of the H atoms forming almost two H···H bonds at 1.15 Å. Concomitantly, the distance in H_2_ is lengthened to 0.76 Å. The Bader charges for the H atom in the H_3_ and H_2_ units and for the Cl atom are +0.16 and +0.014 and –0.48 respectively. In comparison to H_2_Cl the ionicity on both the H and Cl atoms have increased substantially. The plot of the electron localization function (ELF) shown in Fig. 4 shows localized spin paired electron density within the H_3_ ring and in the H_2_ molecule (ELF over 0.8). Weak pairing is also observed between one of the H in the H_3_ ring with the two H atoms of H_2_.

It is tempting to relate the positively charged H_3_ unit to an isolated trihydrogen cation H_3_^+^.

**Fig. 5:**
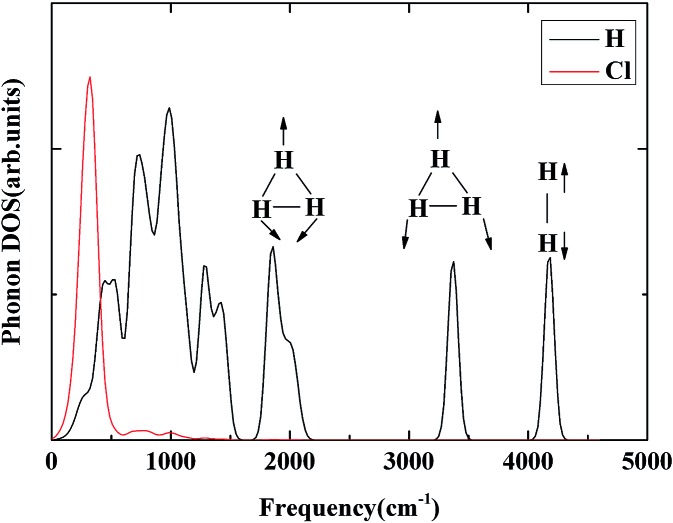
The phonon densities of states for *Cc*-H_5_Cl at 300 GPa are shown. The modes corresponding to the triatomic H_3_^+^ stretch, as well as the H_2_ vibron are denoted.

H_3_^+^ has a perfect triangular structure with H–H bond distance of 0.90 Å. For comparison, at 300 GPa the average intramolecular H–H distance in the H_3_ unit in H_5_Cl is 0.87 Å and a total charge of +0.48 (3 × 0.16). In addition, the H–H distance of 0.76 Å in the H_2_ unit is only slightly perturbed from the isolated molecule. Therefore, it is not unreasonable to suggest that the high pressure H_5_Cl structure is composed of H_3_^+^ stabilized in the solid state through primarily ionic interactions with the Cl atoms and secondary weak interactions with a pair of H_2_ molecules. To investigate further the properties of the H_3_^+^ unit in H_5_Cl, the phonon densities of states calculated at 300 GPa are shown in Fig. 5. The low-energy vibrations from 100–500 cm^–1^ are dominated by the Cl atomic motions. The phonon modes in the region from 1800 to 3400 cm^–1^ can be assigned to H_3_^+^ molecular vibrations and then near 4000 cm^–1^ to the H_2_ molecule. The H_3_^+^ bend vibrations are split into two bands centered at 1800 cm^–1^ and 2000 cm^–1^. The peak at 3350 cm^–1^ is attributed to the stretching vibration. In comparison, the fundamental frequencies for isolated H_3_^+^ are *ν* (stretch) = 3220 × 48 cm^–1^ and *ν* (bend) = 2545 × 99 cm^–1^, the latter is a degenerated mode.^31^ The vibration frequency is 4350 cm^–1^ for a free H_2_ molecule. The main mechanism for the synthesis of H_3_^+^ by experiment is *via* the chemical reaction:^32^ H^+^ + H_2_ → H_3_^+^. The concentration of H_2_^+^ is the limiting factor on the rate of this reaction. H_2_^+^ can only be produced in interstellar space by the ionization of H_2_ by a cosmic ray. In H_5_Cl, the electronegative chlorine atoms acquire electrons from the hydrogen. From previous studies, it is known that high pressure facilitates the transfer of electrons from the more electropositive element into the interstitials of a crystal, forming electrodes. In this case, these electrons, being transferred to the chlorine, are originating from the hydrogen. The formation of multicenter H_3_···H_2_ clusters helps to delocalize the positive charge and stabilizes the system. This explains the unexpected formation of H_3_^+^-like units in the crystal structure.

The nature of the bonding in the group 17 hydrides at high pressure is different from group 1 and 2 and group 14 hydrides. One observed a gradual shift in the chemical interaction from electron transfer in electropositive group 1 and 3 compounds to covalent bonding group 14 and finally the ionic bonding in group 17 elements. Although this study was focused on Cl, we anticipate a similar bonding mechanism is applicable to other halogen hydrides.

## Conclusions

We have investigated the phase stability of the HCl–H_2_ system at high pressure using the PSO algorithm in combination with *ab initio* density functional based electronic calculations. Between 100–300 GPa, two stable phases with the H_5_Cl and H_2_Cl stoichiometries were found. The basic structure of the high pressure phases is similar to the low temperature ambient pressure structure of HCl. H_2_Cl consists of zigzag H–Cl chains and non-interacting H_2_ molecules. The most usual and informative finding is that while the chain like structure is preserved in H_5_Cl, the H atoms connecting the Cl in the chains are replaced by units consisting of weakly interacting H_2_···H_3_. The H_3_^+^ is positively charged and stabilized from the formation of multicenter bonds. The similarity in the local structure, vibrational frequencies and electronic charge compel us to relate the unit to the isolated H_3_^+^ molecule. It is also found that the effect of pressure on the electronic structure of group 17 hydrides is very different from the more electropositive group 1 and 2 elements: the electron-rich Cl atom and anion do not transfer their electrons into interstitial space of the crystal under very high compression. In fact electrons are removed from the H atoms leading to the formation of cationic clusters that benefit from multicenter bonding.

## References

[cit1] Ashcroft N. W. (1968). Phys. Rev. Lett..

[cit2] Hooper J., Zurek E. (2012). Chem.–Eur. J..

[cit3] Zurek E., Hoffmann R., Ashcroft N. W., Oganov A. R., Lyakhov A. O. (2009). Proc. Natl. Acad. Sci. U. S. A..

[cit4] Hooper J., Zurek E. (2012). J. Phys. Chem. C.

[cit5] Wang Z., Yao Y., Zhu L., Liu H., Iitaka T., Wang H., Ma Y. (2014). J. Chem. Phys..

[cit6] Lonie D. C., Hooper J., Altintas B., Zurek E. (2013). Phys. Rev. B: Condens. Matter Mater. Phys..

[cit7] Shamp A., Hooper J., Zurek E. (2012). Inorg. Chem..

[cit8] Hooper J., Altintas B., Shamp A., Zurek E. (2013). J. Phys. Chem. C.

[cit9] Strobel T. A., Goncharov A. F., Seagle C. T., Liu Z., Somayazulu M., Struzhkin V. V., Hemley R. J. (2011). Phys. Rev. B: Condens. Matter Mater. Phys..

[cit10] Li Y., Gao G., Li Q., Ma Y., Zou G. (2010). Phys. Rev. B: Condens. Matter Mater. Phys..

[cit11] Gao G. (2010). Proc. Natl. Acad. Sci. U. S. A..

[cit12] Martin D., McDaniel E., Meeks M. (1961). Astrophys. J..

[cit13] Wang Y., Lv J., Zhu L., Ma Y. (2010). Phys. Rev. B: Condens. Matter Mater. Phys..

[cit14] Wang Y. Lv J. Zhu L. Ma Y., Comput. Phys. Commun., 2012, 183 , 2063 –2070 , , CALYPSO code is free for academic use, please register at http://www.calypso.cn .

[cit15] Li P., Gao G., Wang Y., Ma Y. (2010). J. Phys. Chem. C.

[cit16] Lv J., Wang Y., Zhu L., Ma Y. (2011). Phys. Rev. Lett..

[cit17] Zhu L., Wang H., Wang Y., Lv J., Ma Y., Cui Q., Ma Y., Zou G. (2011). Phys. Rev. Lett..

[cit18] Guillaume C. L., Gregoryanz E., Degtyareva O., McMahon M. I., Hanfland M., Evans S., Guthrie M., Sinogeikin S. V., Mao H. K. (2011). Nat. Phys..

[cit19] Zhu L., Wang H., Wang Y., Lv J., Ma Y., Cui Q., Ma Y., Zou G. (2011). Phys. Rev. Lett..

[cit20] Perdew J. P., Burke K., Ernzerhof M. (1996). Phys. Rev. Lett..

[cit21] Kresse G., Furthmüller J. (1996). Phys. Rev. B: Condens. Matter Mater. Phys..

[cit22] Monkhorst H. J., Pack J. D. (1976). Phys. Rev. B: Solid State.

[cit23] Bader R. F. W. (1985). Acc. Chem. Res..

[cit24] Henkelman G., Arnaldsson A., Jónsson H. (2006). Comput. Mater. Sci..

[cit25] Tang W., Sanville E., Henkelman G. (2009). J. Phys.: Condens. Matter.

[cit26] Klimeš J., Bowler D. R., Michaelides A. (2011). Phys. Rev. B: Condens. Matter Mater. Phys..

[cit27] Parlinski K., Li Z. Q., Kawazoe Y. (1997). Phys. Rev. Lett..

[cit28] Togo A., Oba F., Tanaka I. (2008). Phys. Rev. B: Condens. Matter Mater. Phys..

[cit29] Ma Y., Tse J. S. (2007). Solid State Commun..

[cit30] Sándor E., Farrow R. (1967). Nature.

[cit31] Carney G. D. (1980). Mol. Phys..

[cit32] Hogness T. R., Lunn E. G. (1925). Phys. Rev..

